# Antibodies against a Surface Protein of *Streptococcus pyogenes* Promote a Pathological Inflammatory Response

**DOI:** 10.1371/journal.ppat.1000149

**Published:** 2008-09-12

**Authors:** Fredrik Kahn, Matthias Mörgelin, Oonagh Shannon, Anna Norrby-Teglund, Heiko Herwald, Anders I. Olin, Lars Björck

**Affiliations:** 1 Department of Clinical Sciences, Division of Infection Medicine, Lund University, BMC, B14, Lund, Sweden; 2 Karolinska Institute, Center for Infection Medicine, Karolinska University Hospital Huddinge, Stockholm, Sweden; Children's Hospital Boston, United States of America

## Abstract

Streptococcal toxic shock syndrome (STSS) caused by *Streptococcus pyogenes* is a clinical condition with a high mortality rate despite modern intensive care. A key feature of STSS is excessive plasma leakage leading to hypovolemic hypotension, disturbed microcirculation and multiorgan failure. Previous work has identified a virulence mechanism in STSS where M1 protein of *S. pyogenes* forms complexes with fibrinogen that activate neutrophils to release heparin-binding protein (HBP), an inducer of vascular leakage. Here, we report a marked inter-individual difference in the response to M1 protein–induced HBP release, a difference found to be related to IgG antibodies directed against the central region of the M1 protein. To elicit massive HBP release, such antibodies need to be part of the M1 protein–fibrinogen complexes. The data add a novel aspect to bacterial pathogenesis where antibodies contribute to the severity of disease by promoting a pathologic inflammatory response.

## Author Summary

*Streptococcus pyogenes*, also known as group A streptococcus, is a common bacterial pathogen mostly causing rather harmless infections such as pharyngitis (“strep throat”) and impetigo (a superficial skin infection). However, sometimes these bacteria give rise to severe invasive infections, and one of the most feared conditions in infection medicine is streptococcal toxic shock syndrome (STSS). In STSS, the mortality remains high despite modern intensive care and the fact that *S. pyogenes* is susceptible to penicillin. Why some people develop this serious condition and others not is still not fully understood. The common dogma is that antibodies against a pathogenic microorganism are protective, but in this work we show that certain antibodies actually increase the risk for a more severe disease. This information suggests that these antibodies could be used as therapeutic targets, and the results may also facilitate the identification of individuals prone to developing STSS.

## Introduction

*Streptococcus pyogenes* is a major human bacterial pathogen that causes a wide range of infections from common and mostly uncomplicated cases of pharyngitis and impetigo, to severe invasive infections [1]. Since the 1980s an unexplained increased incidence of these severe infections has been reported world-wide, and it is estimated that invasive *S. pyogenes* infections, streptococcal toxic shock syndrome (STSS) and necrotizing fasciitis, are responsible for more than 150 000 deaths annually [2],[3]. These conditions have attracted considerable attention and concern, also in the public, due to their acute and dramatic nature. Despite modern intensive care and prompt and adequate antibiotic therapy, they are associated with high mortality rates [4]–[6].

A potent inflammatory response leading to shock and organ failure is typical of STSS. Previous work has shown that secreted *S. pyogenes* exotoxins play an important role in STSS by inducing this response. Thus, STSS patients have a propensity to produce high levels of inflammatory cytokines in response to these superantigens [7]. There is also a correlation between the severity of *S. pyogenes* infection and HLA class II allelic variation [8], suggesting that the regulation of superantigen-triggered cytokine response contributes to *S. pyogenes* pathogenicity. Moreover, binding of plasminogen to the bacterium and the subsequent activation to plasmin represents a virulence mechanism that has been proposed to promote the transition from a localized infection into a severe invasive disease [9]. Streptolysin-O-induced platelet/neutrophil aggregation has also been suggested to contribute to the vascular dysfunction in severe *S. pyogenes* infection [10].

A key symptom in STSS is a massive vascular leakage (these patients often require 10–20 liters of intravenous fluid per day) leading to hypovolemic hypotension and multiorgan failure. A molecular mechanism contributing to the vascular leakage in STSS has been identified [11]. M protein, a classical virulence determinant of *S. pyogenes* (for references, see review [12]), is released from the surface of *S. pyogenes*, forms complexes with fibrinogen that activate neutrophils to secrete Heparin-Binding Protein, HBP (also known as azurocidin or CAP37), a powerful inducer of increased vascular permeability [13]. The finding that M protein binds fibrinogen was first reported by Kantor et al [14], and the binding was subsequently mapped to the NH_2_-terminal half of the protein but excluding the most distal hypervariable region [15]. The proinflammatory effect of M1 protein-fibrinogen complexes was further underlined by a recent and elegant investigation, showing that a mutated form of M1 protein with lower affinity for fibrinogen, lost its capacity to induce HBP release and vascular leakage [16]. In the present work, a marked inter-individual difference was recorded for the M protein-induced HBP release in the blood of healthy individuals. This difference was explained by the presence of specific IgG antibodies against M1 protein, giving rise to protein complexes containing M1 protein, fibrinogen and IgG. Such complexes induce a massive release of HBP by the simultaneous activation of β_2_ integrins (via fibrinogen) and IgGFc receptors (via IgG bound to M protein in the complexes exposing their Fc regions). The finding that IgG antibodies against a bacterial antigen participate in the induction of a severe and pathologic inflammatory response, represents a novel concept in bacterial virulence.

## Results

### Individual variation in the M1 protein-induced release of HBP in human blood is due to IgG antibodies

**Figure 1:**
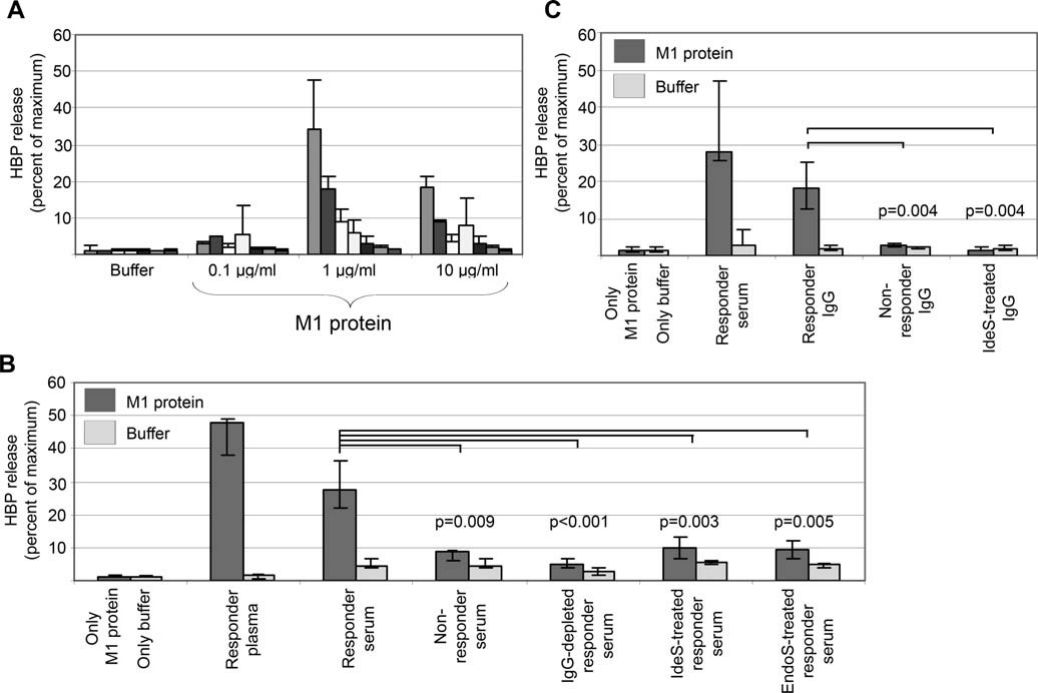
M protein-induced release of HBP shows inter-individual variation which is dependent on IgG antibodies. (A) Buffer or various amounts of M1 protein was incubated with whole blood (diluted 1∶10) from seven healthy individuals. The release of HBP was measured and expressed as percentage of maximum (total lysis of blood with Triton-X). Data from at least three separate experiments are shown and expressed as the mean±SD. (B) Whole blood (diluted 1∶10) from an individual not responding with HBP release to the addition M1 protein, was used. M1 protein (dark grey bars) or buffer (light grey bars), alone or together with plasma or serum samples from a responding or a non-responding individual, was incubated with the blood. Serum from the responder was also depleted from IgG by protein G absorption, pre-treated with IgG-cleaving proteinase IdeS or with the endoglycosidase EndoS to remove the glycan from IgG, prior to the incubation. Data are the median±interquartile range from at least four different experiments. The HBP release elicited by serum from non-responders and pre-treated sera were compared against serum from the responder using the Mann-Whitney U test and the result is shown as p values uncorrected for multiple comparisons. (C) Whole blood (diluted 1∶10) from an individual not responding with HBP release to M1 protein, was used. M1 protein (dark grey bars) or buffer (light grey bars), alone or together with responder serum or purified IgG from a responding or a non-responding individual, was added the blood. Where indicated IgG from the responder was pre-treated with IdeS. Data are the median±interquartile range from at least four different experiments. The HBP release elicited by responder IgG was compared with the response elicited by non-responder IgG and by responder IgG pre-treated with IdeS using the Mann-Whitney U test showing the crude p-values before Bonferroni correction.

As mentioned, previous work has shown that M1 protein when added to human blood, forms complexes with fibrinogen which activate neutrophils to release HBP [11].The starting point for this study was the observation that there is a marked individual variation in the response to M1 protein. As shown in Fig. 1A, the blood of some individuals responds with massive HBP release, whereas others are non-responders (defined as a response of less than 10% release of the total amount of HBP). As previously reported [11], maximum HBP release in responder blood was obtained at an M1 protein concentration of 1 µg/ml in blood diluted 1∶10. The recombinant M1 protein used in these experiments does not contain LPS at a concentration that influences the HBP release [11].

The antibody titers against M1 protein were determined in serum samples from 19 responders and non-responders (Table 1). Unexpectedly, the individuals with powerful responses also had high anti-M1 antibody titers. When serum from a responder was added to the blood of a non-responder together with M1 protein, the serum induced HBP release. However, serum from a non-responder did not (Fig. 1B). The IgG antibodies in the responder serum were removed by protein G-Sepharose absorption, and this IgG-depleted serum could no longer induce HBP release when added to non-responder blood (Fig. 1B). The role of IgG was further investigated by the use of enzymes that have IgG as their only substrate. IdeS, also called Mac-1 [17], is a cysteine proteinase secreted by *S. pyogenes* which is highly specific for IgG antibodies [18]. The enzyme cleaves IgG in the hinge region to generate one F(ab')_2_ fragment and the two heavy chain components of the Fc fragment. EndoS is an endoglycosidase, also from *S. pyogenes*, that specifically removes the glycan from the Fc region of IgG [19]. Responder serum was pre-treated with either IdeS or EndoS, and then separately added (together with M1) to non-responder blood. These serum samples lost their capacity to induce HBP release in non-responder blood (Fig. 1B). Finally, IgG was purified from responder and non-responder serum. Only IgG from responder serum could, when added to non-responder blood, induce HBP-release in the presence of M1 protein, and following pre-treatment with IdeS, IgG lost its HBP-releasing activity (Fig. 1C). Taken together the results demonstrate that IgG antibodies are involved in the activation of neutrophils leading to HBP release. IgG F(ab')_2_ fragments generated by IdeS cleavage and EndoS treated IgG devoid of its glycan, still recognize and bind their antigen. However, the Fc region is cleaved off by IdeS [18] and removal of the glycan by EndoS results in lower affinity for the Fc receptor [20]. This suggests that the IgG antibodies in responder serum inducing HBP release require an intact Fc region to activate the neutrophils, presumably by interacting with IgGFc receptors.

**Table 1:** HBP release is related to anti-M1 titers.

Donor	HBP release (% of maximum)	Anti-M1 titer (ELISA-index)
**1**	1	4
**2**	1	9
**3**	1	1
**4**	1	4
**5**	2	3
**6**	3	10
**7**	3	39
**8**	4	13
**9**	5	20
**10**	6	59
**11**	8	40
**12**	8	60
**13**	9	79
**14**	15	65
**15**	17	44
**16**	18	129
**17**	18	75
**18**	34	174
**19**	34	96

### M1 protein and anti-M1 IgG antibodies induce HBP release in human blood

**Figure 2:**
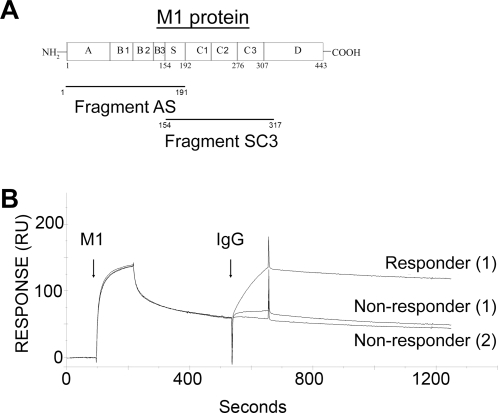
Surface plasmon resonance spectroscopy detects high affinity IgG antibodies against M1 protein in responder serum. (A) Schematic depiction of mature M1 protein without the signal peptide showing the AS and SC3 fragments. Fibrinogen binding is mediated by the B domains, and the protein is associated with the bacterial cell wall through the COOH-terminal D region. Numbers refer to amino acid residue positions. (B) M1 protein was flushed over BIAcore chips coated with fibrinogen. The dissociation phase of this interaction was followed by the injection of purified IgG preparations from one responder and two non-responders, and the binding is expressed in resonance units (RU).

The observation that IgG antibodies are required to induce a powerful HBP release, raised questions about the specificity of these antibodies. M1 protein, schematically depicted in Fig. 2A, appeared as a possible candidate antigen, and surface plasmon resonance technology was used to test this hypothesis. In these experiments, M1 protein was initially flushed over a surface coated with fibrinogen (Fig. 2B). The affinity between M1 protein and fibrinogen is high, 1.1×10^10^ M^−1^ [21], which is evident from the rapid binding of M1 protein in the left part of Fig. 2B. The interaction between fibrinogen and M1 protein was allowed to reach equilibrium, followed by separate injections of IgG purified from the sera of one responder and two non-responders. In contrast to the IgG preparations from the non-responders, responder serum contains IgG antibodies that bind to the M1 protein associated with fibrinogen (Fig. 2B, right part). These results indicate that IgG antibodies inducing HBP release in responder sera are directed against the M1 protein.

**Figure 3:**
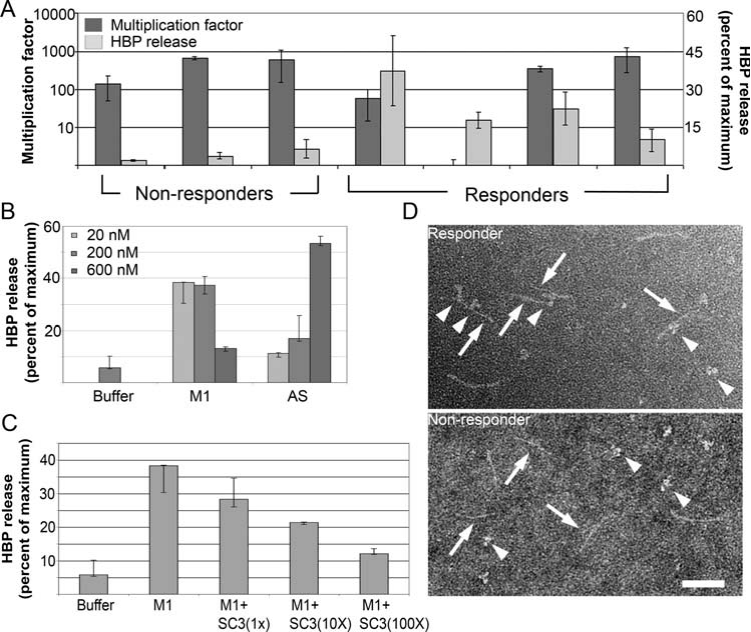
Mapping of the antigenic region(s) in M1 protein responsible for HBP release. (A) Bacteria were added to whole blood from seven donors; three non-responders and four responders. After three hours of incubation, blood was plated out and the number of colony forming units was determined. In addition, the amount of HBP was determined in the blood from the seven donors. The multiplication factors and the HBP values represent the mean±SD of at least three different experiments. (B) Whole blood (diluted 1∶10) from an individual not responding with HBP release to M1 protein, was used. Buffer or different concentrations of M1 protein or fragment AS of M1, was incubated with the blood together with serum from a responder. Data are the median±interquartile range from at least four different experiments. (C) Whole blood (diluted 1∶10) from an individual not responding with HBP release to M1 protein was used. M1 protein was added to the blood (1 µg/ml corresponding to 20 nM) supplemented with responder serum, together with 1, 10 or 100 times molar excess of fragment SC3 of M1. Data are the median±interquartile range from at least four different experiments. (D) Negative staining and electron microscopy of M1 protein (arrows) incubated with IgG antibodies (arrow heads) purified from the serum of a responder or a non-responder. Scale bar: 50 nm.

A classical observation in streptococcal research is that *S. pyogenes* bacteria survive and grow in human whole blood, provided there are no type specific anti-M protein antibodies directed against the hyper-variable NH_2_-terminal region of the M protein expressed by the strain in question [22]. This region in the very NH_2_-terminal region of M proteins comprises 50–100 amino acid residues and shows a high degree of variation among strains of different M types. There are more than one hundred M types [23], but within a given type the sequence is conserved. To find out whether antibodies against this NH_2_-terminal region are important for the induction of HBP release, the growth of M1 bacteria was investigated in the blood of seven different individuals, four responders and three non-responders. The results of Fig. 3A show that the bacteria grew well in the blood from the non-responder and three of the responders, whereas growth was completely inhibited in the blood of one responder. The lack of correlation between the presence of bactericidal antibodies directed against the NH_2_-terminal region of M1 protein and HBP release, indicates that IgG antibodies against other regions of the M1 protein are required for HBP release. The AS fragment covers the 191 NH_2_-terminal amino acids of the mature protein M1 protein containing the hypervariable A domain , the fibrinogen-binding B domains and the S domain (Fig. 2A). Adding the AS peptide to blood from a non-responder together with serum from a responder, elicited HBP release (Fig. 3B). Compared to intact M1 protein the dose response is different for the AS fragment. This has previously been reported [11] and is due to the fact that different molar concentrations of M1 protein and the AS fragment are required to generate protein complexes in plasma. In contrast to AS, the SC3 fragment (Fig. 2A) does not contain the fibrinogen-binding B domains or the hypervariable region (A), but spans the S domain and the COOH-terminal region of the mature protein. This peptide did not induce HBP release in an identical experiment, suggesting that the interaction with fibrinogen is required. However, when added at a molar excess together with M1 protein to the blood of a non-responder supplemented with responder serum, HBP release was inhibited (Fig. 3C). This indicates that IgG antibodies promoting HBP release are absorbed by binding to the SC3 fragment. Thus, since the only domain shared by the AS and the SC3 fragments is the S domain and the SC3 fragment inhibits the response, we conclude that the IgG antibodies necessary for a massive HBP response bind to this domain (this is also supported by the electron micrograph in Figure 3D). In order to further pin-point the interaction, a synthetic peptide covering the S domain was produced and tested for its capacity to inhibit the response. This peptide had no inhibitory activity. Given that short peptides often do not fold properly this was not an unexpected result. The response to two other M proteins, M3 and M5 protein, was also investigated in blood from M1 responders. These proteins were tested under the same conditions as the M1 protein, but failed to elicit HBP release (data not shown). Finally, IgG antibodies isolated from responder and non-responder serum were separately incubated with purified M1 protein, and the resulting protein complexes were analyzed by negative staining electron microscopy (Fig. 3D). Inspection of large fields revealed that compared to IgG from non-responders, responder IgG generated much more complexes (data not shown). In these complexes, the antibodies predominantly bound to epitopes in the central region of the M1 protein (Fig. 3D). Taken together, the experiments described in Fig. 3A–D suggest that the IgG antibodies eliciting HBP release in responder serum, are directed against epitopes within or close to the central S domain of the M1 protein.

### Protein complexes containing M1 protein, anti-M1 IgG antibodies, and fibrinogen activate neutrophils to release HBP

**Figure 4:**
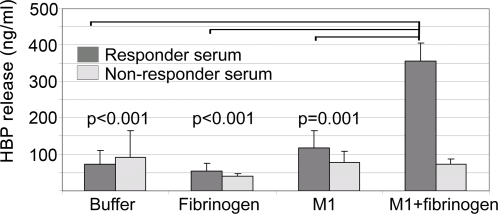
Fibrinogen is required for M1 protein-induced HBP release by purified neutrophils. Fibrinogen (150 µg), M1 protein (0.5 µg), or fibrinogen (150 µg) plus M1 protein (0.5 µg) were dissolved in 0.45 ml PBS, followed by the addition of 50 µl responder (filled bars) or non-responder serum (open bars). Neutrophils (2×10^5^ cells) were added and after incubation for 30 min at 37°C, cells were spun down and the amount of HBP was determined in the supernatants. The figure shows the mean±SD of three separate experiments. The data obtained with responder serum were compared using one way analysis of variance, and the results showed a statistically significant difference (p<0.001). The results obtained with M1 protein+fibrinogen were compared with the HBP release generated by buffer, fibrinogen or M1 protein alone, using the Bonferroni corrected t-test. The corresponding p-values are indicated.

**Figure 5 ppat-1000149-g005:**
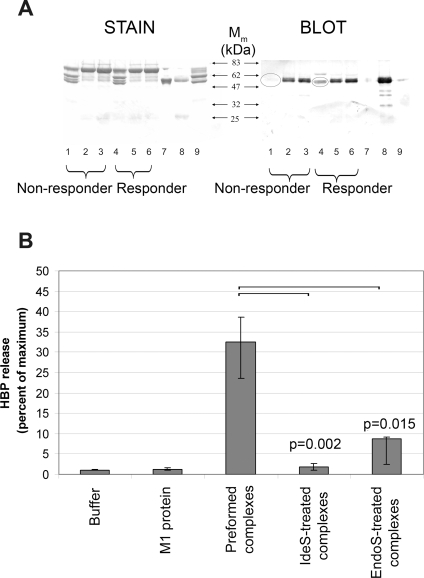
M1 protein forms HBP-releasing precipitates with fibrinogen and IgG in responder plasma. (A) Purified M1 protein was added (10 µg/ml) to the plasma of a responder or a non-responder (diluted 1∶10). After incubation, the resulting precipitates were spun down and the supernatants were saved. Precipitates were washed, boiled in sample buffer and subjected to SDS-PAGE (lanes 1 and 4). The supernatants (see above) were run in lanes 2 and 5, human plasma diluted 1∶500 was run in lanes 3 and 6, M1 protein (1.2 µg) in lane 7, human polyclonal IgG (2 µg) in lane 8, and human fibrinogen (2.6 µg) in lane 9. One gel was stained with Commassie Blue (STAIN), whereas an identical gel was blotted onto an Imobilon filter and probed with goat antibodies against human IgG (BLOT). (B) Whole blood diluted 1∶10 from a non-responder was used. Buffer or M1 protein (1 µg/ml) was added. M1 protein was pre-incubated with responder plasma and the complexes formed were also tested. Such complexes were also digested with IdeS or EndoS and subsequently added to the blood. The release of HBP was measured and the data shown represent the median±interquartile range from six separate experiments. The HBP release from blood supplemented with complexes was compared to the IdeS- or the EndoS- treated complexes using the Mann-Whitney U test, and the result is shown as p values uncorrected for multiple comparisons.

**Figure 6:**
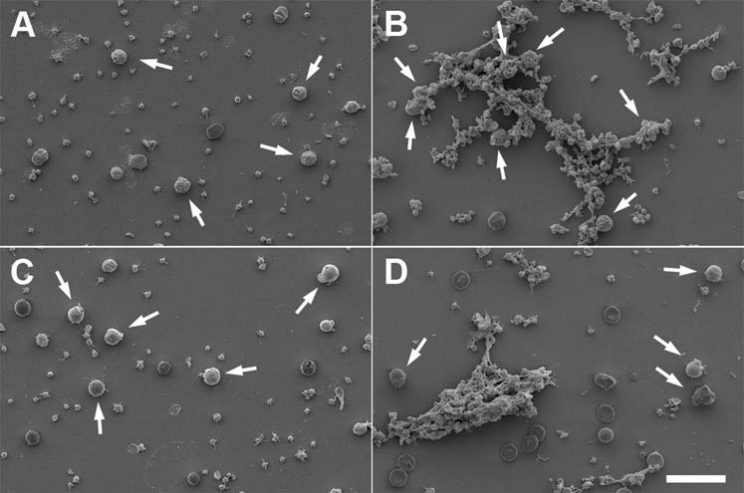
M1 protein-fibrinogen-IgG complexes bind to neutrophils/monocytes in responder blood. Buffer (A and C) or M1 protein (1 µg/ml) (B and D) was incubated with whole blood (diluted 1∶10) from a responder (A and B) and a non-responder (C and D). Red blood cells were lysed and the samples were subjected to scanning electron microscopy. Arrows indicate neutrophils/monocytes. Scale bar: 20 µm.

In a series of experiments, summarized in Fig. 4, it was demonstrated that a powerful release of HBP by purified neutrophils is generated only in the presence of M1 protein, responder serum, and fibrinogen. The results suggest that protein complexes comprised of M1 protein, IgG antibodies against M1 protein, and fibrinogen, are required, and that ordinary antigen-antibody immune complexes are not sufficient to induce HBP-release. To challenge this hypothesis, purified M1 protein was added to responder and non-responder plasma. The precipitates formed were analyzed by SDS-PAGE and Western blotting (Fig. 5A). Precipitates from responder plasma contained all three proteins, whereas IgG was missing in precipitates formed by fibrinogen and M1 protein in non-responder plasma. M1 protein also binds albumin [24], which explains the band of 65–70 kDa seen in both precipitates. To investigate if the precipitates contained complement proteins, Western blotting was performed with anti-C3 antibodies. No significant bands could be detected in any of the precipitates (data not shown). When added to whole blood from a non-responding individual, pre-formed M1 protein precipitates generated in responder plasma, induced HBP release (Fig. 5B). As shown in Fig. 5B, this activity is abolished when the precipitates are treated with IdeS or EndoS, enzymes which specifically act on the Fc region of IgG. Moreover, non-responder serum does not elicit HBP release. Finally, M1 protein was added to responder or non-responder blood to induce the formation of protein aggregates [11], which were subsequently analyzed by scanning electron microscopy (Fig. 6). The protein aggregates formed in responder blood contained neutrophils/monocytes, whereas such cells were absent in aggregates of non-responder blood. The HBP release was also measured in these blood samples incubated with M1 protein, and elevated HBP levels were only recorded in responder blood (data not shown). The results described in this paragraph and in Figures 4–6, show that protein complexes containing M1 protein, fibrinogen, and anti-M1 protein IgG antibodies, are required for a massive HBP release.

### HBP release, Fcγ receptors, and the role of platelets

**Figure 7:**
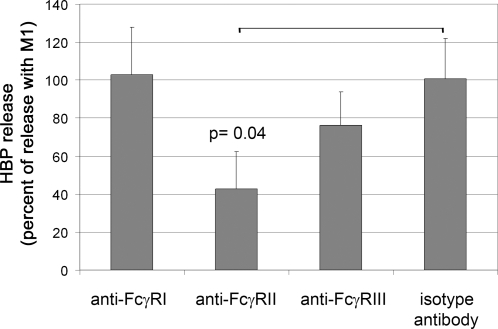
Antibodies against FcγRII inhibit HBP release. Whole blood (diluted 1∶10) was incubated with antibodies against different FcγRs, isotype control antibodies, or buffer alone, for 20 minutes at 37°C. M1 protein was added and the HBP release was measured. Data are expressed as percentage of release without any antibodies added, and are the mean±SD from three different individuals, each done in duplicate. The different treatment groups were compared using ANOVA, and when found significantly different the treatment groups were compared against the control group using Dunnett's 2 sided test.

To investigate the significance of Fcγ receptor binding for HBP release, inhibition experiments were performed with antibodies against the various Fc receptors present on neutrophils (for references see reviews [25],[26],[27]). Responder blood was pre-incubated with anti-FcγRI, anti-FcγRII, anti-FcγRIII, or isotype control antibodies for 20 minutes, followed by the addition of M1 protein. Significant blocking of HBP release was obtained only with the antibodies against FcγRII (Fig. 7).

It was previously demonstrated that M1 protein forms complexes with fibrinogen and IgG that activate platelets [28], and we therefore investigated whether activated platelets through interactions with neutrophils contribute to the release of HBP. The results suggest that the activation of neutrophils resulting in HBP release is independent of platelets. Thus, neutrophils purified from platelets retained their full HBP-releasing activity when stimulated by M1 protein in the presence of responder plasma (data not shown).

### Responder serum aggravates M1 protein-induced lung lesions in mice

**Figure 8:**
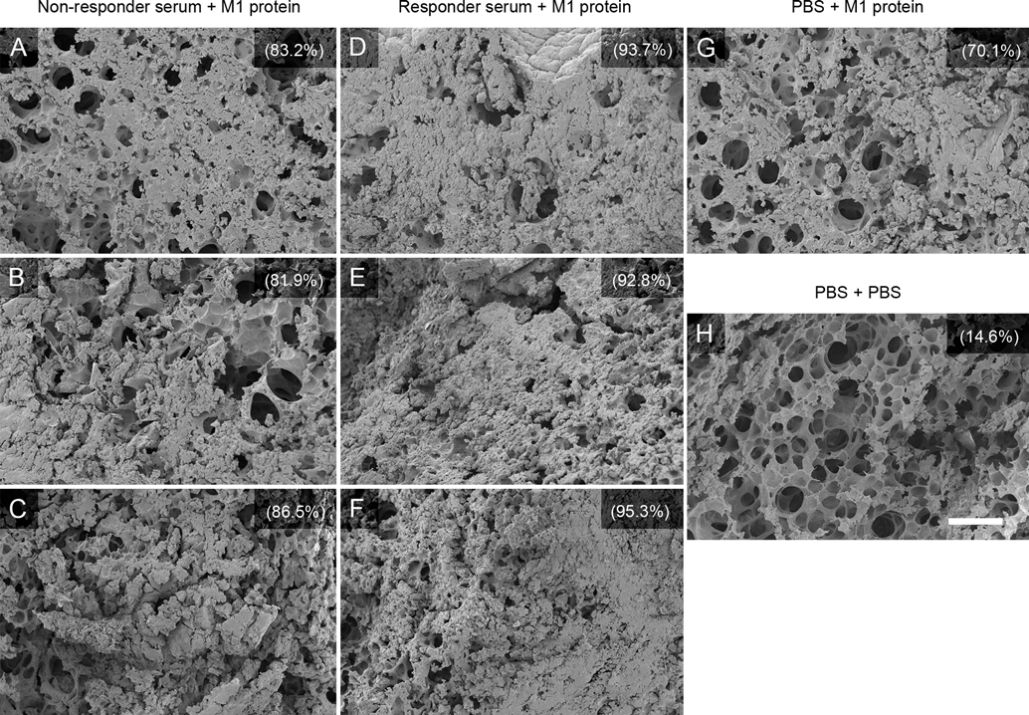
Lung pathology of mice injected with M1 protein and human serum. Balb/c mice were injected i.p. with serum from a non-responder (3 mice, 1–3) or a responder (3 mice, 4–6), or with PBS (2 mice, 7–8). Twenty hours later the serum treated mice and one of the PBS treated animals were injected i.v. with M1 protein, whereas the remaining mouse injected with PBS i.p. received PBS i.v. After another 4 hours the animals were sacrificed and their lungs were analyzed by scanning electron microscopy. Panel A–C: lung tissue from the mice (1–3) treated with non-responder serum plus M1 protein. Panel D–F: lung tissue from the mice (4–6) treated with responder serum and M1 protein. Panel G: lung tissue from the mouse (7) that received PBS and M1 protein. Panel H; lung tissue from the mouse receiving buffer alone. Each lung tissue sample was evaluated from 30 different fields covering an entire lung section and the percentage fields exhibiting hemorrhage and fibrin deposits is indicated for the 8 samples. Lung tissue samples from the animals that received responder serum and M1 protein (D–F), could be blindly distinguished from the other samples.

Intravenous (i.v.) injection of M1 protein induces lung lesions in mice characterized by hemorrhage, fibrin deposits, and alveolar swelling [11]. To address the question whether responder antibodies influence this effect of M1 protein, mice were injected i.p. with responder serum, non-responder serum, or PBS, followed by i.v. injection of M1 protein (15 µg/animal) or PBS 20 hours later. After another 4 hours the animals were sacrificed and the lungs were removed and analyzed by scanning electron microscopy. As previously reported, lesions with hemorrhage, fibrin deposits, and alveolar swelling were found in all mice injected with M1 protein, but they were more severe and wide-spread in the lungs of mice that received responder-serum (Fig. 8). To avoid a possible effect of LPS, the M1 protein preparation used in these experiments was purified from *S. pyogenes*. Since mice injected with only M1 protein also exhibit lung lesions, the blood of these animals were screened by ELISA for anti-M1-antibodies. No such antibodies were detected (data not shown). The results suggest that the combination of responder serum and M1 protein causes more severe lung lesions in mice than M1 protein alone.

### Complexes of M1 protein, IgG and fibrinogen are formed in a patient with necrotizing fasciitis and streptococcal toxic shock syndrome (STSS)

**Figure 9:**
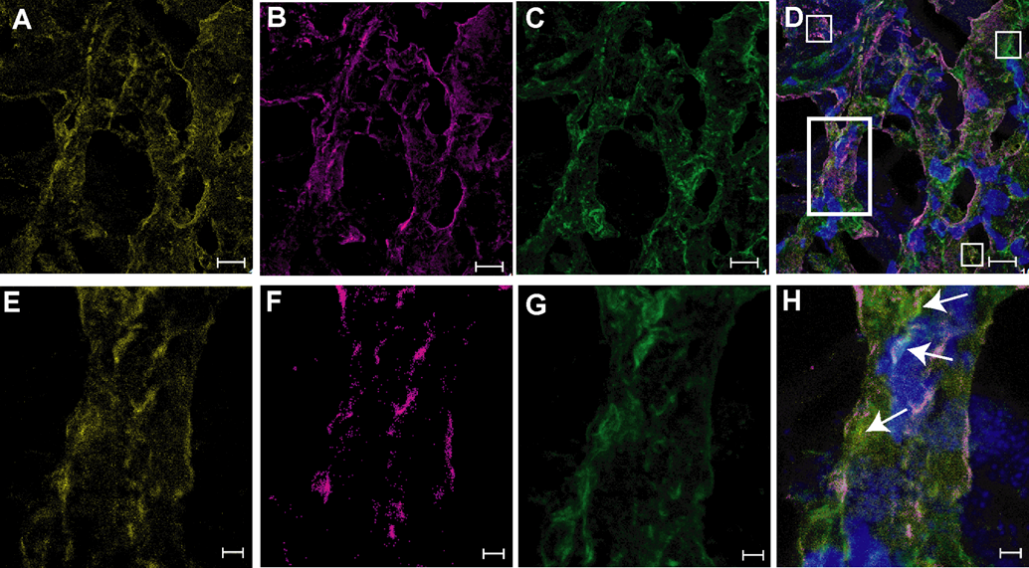
Extensive formation of M1 protein-fibrinogen-IgG complexes is detected at the local site of *S. pyogenes* tissue infection. A tissue biopsy obtained from a patient with necrotizing fasciitis and streptococcal toxic shock syndrome caused by an M1 strain, was sectioned, fixed, and stained for the M1 protein, fibrinogen and IgG as detailed in Methods. The stainings were analyzed by confocal microscopy using a 63× oil objective lens. The figure shows simulated maximum projections of sequential scans at two different magnifications; panel A–D, scale bar = 10 µm and in panel E–H, scale bar = 2 µm. A and E: M1 protein (Alexa 546, yellow), B and F: fibrinogen (Alexa 633, magenta), C and G: IgG (Alexa 488, green), D and H: overlay. Despite a high degree of co-localization, each marker could be identified also individually (indicated by white squares in D). The area indicated by the white rectangle in D was chosen for re-analysis at a higher magnification, panel E–H. The arrows in H indicate areas of co-localized M1 protein, fibrinogen and IgG. Cellular infiltrates are shown in blue by the nuclear staining, dapi.

We have previously identified complexes of fibrinogen and M1 protein in the affected tissue of a patient with necrotizing fasciitis and STSS caused by an M1 isolate of *S. pyogenes* [11]. Here we extend these studies to also include assessment of IgG antibodies in the tissue. By use of protein L, a bacterial protein which binds human immunoglobulins with high and specific affinity [29], complexes of fibrinogen, M1 protein and IgG were visualized at the epi-center of the tissue infection (Fig. 9). There were also areas with only M1 protein, fibrinogen or IgG, verifying the specificity of the stainings (Figure 9D). Some of the M1 protein was associated with the streptococcal surface, but most of the protein was found in the tissue not associated with the bacteria and in complexes with fibrinogen (Fig. 9). IgG detected by protein L was found dispersed within the tissue as well as in the M1 protein-fibrinogen complexes. The data show the *in vivo* presence of M1-fibrinogen-IgG complexes in the tissue of a patient with necrotizing fasciitis and STSS.

## Discussion

The outcome of a bacterial infection depends on the molecular interactions between the bacterium and the human host. *S. pyogenes* has developed a multitude of strategies to evade human defenses, and it interferes with and manipulates the immune, complement, coagulation and contact systems, whereby inflammatory reactions can be induced, enhanced, down-regulated or inhibited (for references see reviews [1],[30]). The vast majority of individuals that are colonized by *S. pyogenes* are asymptomatic carriers, but when symptoms occur pronounced inflammation is typical for *S. pyogenes* infections, also in local infections such as pharyngitis, pharyngo-tonsillitis, and impetigo. However, STSS represents an invasive disease where a systemic and extreme inflammation leads to hypovolemic hypotension, disturbed microcirculation and multiorgan failure. It is one of the most serious and feared conditions in infection medicine.

STSS was originally described in the 1980s [31],[32], and since then there has been a worldwide increase in the incidence of the disease [2]. A massive vascular leakage is a hallmark of STSS, which explains the extreme loss of intravascular fluid and the rapid course of the disease. Neutrophil activation is fundamental for the induction of vascular leakage [33], and HBP is solely responsible this effect [13]. The significance of HBP for vascular leakage in severe infectious diseases is emphasized by work in our laboratory showing that elevated plasma levels of HBP is an early indicator of circulatory failure in sepsis (Linder *et al.*, submitted for publication). M protein is a major surface protein of *S. pyogenes* [12], but substantial amounts of M1 protein (0.4–0.6 mg/L growth medium) is also shedded from the bacterial surface [21], suggesting that high local concentrations of M1 protein can be reached at the site of infection. The demonstration that soluble M1 protein forms complexes with fibrinogen that activate neutrophils to unload their HBP content, provided one explanation for how *S. pyogenes* induces vascular leakage [11]. There are more than a hundred different M serotypes [23], but strains of the M1 and M3 serotypes are responsible for approximately fifty percent of the STSS cases [34]. From a patho-physiological point of view, it is therefore noteworthy that the M1 and M3 proteins both contain multiple fibrinogen-binding B domains (see Fig 2A), making it possible for these proteins to generate neutrophil-activating complexes in plasma environment.

When injected i.v. into mice, M1 protein/fibrinogen complexes induce vascular leakage and lung lesions [11],[16]. *S. pyogenes* is regarded as an exclusive human pathogen and in this study we could not identify any pre-existing antibodies against M1 protein in mice, showing that such antibodies are not necessary to cause these lung lesions. Whether the injection of M1 protein i.v. in corresponding amounts into a human non-responder would result in vascular leakage, is of course impossible to test. The fact that M1 itself gives rise to lung lesions in our mouse model, complicates experiments aimed at investigating the pathogenic role of anti-M1 antibodies. However, the fact that our most experienced electron microscopist blindly could identify the most severely affected lungs as those from mice treated with responder serum, supports that such antibodies aggravate the disease. This assumption is also supported by the observation that among 19 tested individuals, only those with significant anti-M1 IgG antibodies were HBP responders.

Several previous investigations have demonstrated that large antigen-antibody complexes generated *in vitro* are capable of activating neutrophils[35],[36],[37]. In this study we find that a massive release of HBP requires not only complexes of M1 protein and anti-M1 IgG antibodies; they must also comprise fibrinogen. M1/fibrinogen complexes activate neutrophils through multiple interactions between fibrinogen molecules in the complexes and β_2_ integrins at the neutrophil surface, and this activation is blocked by a β_2_ integrin antagonist [11]. A major conclusion of this work is that the presence of anti-M1 IgG antibodies in the M1/fibrinogen complexes results in the activation of FcγII receptors, the dominant activating receptors of neutrophils [25]. This dual receptor activation is necessary for a powerful HBP release, an observation that supports recent findings that co-stimulation of neutrophil receptors has a synergistic effect on neutrophil activation [38]. This is also supported by the finding that the M3 and M5 proteins, which both bind fibrinogen but lack a region homologous to the S domain, fail to induce HBP release in M1-responders.

The initial observation that stimulated the current investigation, was the finding that M1 protein, when added to the blood of healthy donors, gave rise to an HBP release that showed a significant inter-individual variation. A central question in infection medicine is why some individuals colonized by a pathogen show no symptoms, whereas other may develop severe disease. As discussed above, this is a highly relevant question concerning the pathogenesis of *S. pyogenes*, where the bacterium is isolated from asymptomatic carriers as well as from patients with a wide range of clinical manifestations, including life-threatening STSS. Previous work in the field has helped to clarify this enigma by demonstrating that STSS patients respond to *S. pyogenes* exotoxins with high levels of pro-inflammatory cytokines [7], and that this response is correlated with certain MHC class II haplotypes [8]. The present work provides an additional explanation to the inter-individual disease susceptibility, by showing that also the humoral immune status contributes to the inflammatory response in STSS.

In viral infections such as Dengue fever, re-infection induces a more severe disease due to preformed IgG antibodies binding to the virus particles. These antibodies expose their Fc regions and activate FcγR-expressing cells, referred to as antibody-dependant enhancement (ADE) of infection [39], which results in a pro-inflammatory state. Such pro-inflammatory mechanisms have previously not been shown to contribute to the pathogenesis of bacterial infections, and fibrinogen has not been ascribed a role in the pathogenesis of Dengue fever. However, an early investigation in a chicken model of *S. aureus* synovitis showed that chickens treated with sera from chickens immunized with *S. aureus*, developed a more severe disease [40]. The molecular background for this was not explained.

The results of this study indicate that individuals with IgG antibodies against the central region of the M1 protein, are more prone to develop vascular leakage and severe disease. It is therefore of interest that patients with fatal STSS and bacteremia caused by *S. pyogenes* strains of the M1 serotype, were reported to have low antibody titers against M1 protein [41]. The serum samples from these patients were taken after the onset of the disease, and the present results indicate that the low titers are due to absorption of anti-M1 IgG antibodies in the M1-fibrinogen-IgG complexes. To challenge this hypothesis, it would be most interesting to analyze a collection of serum samples from individuals prior to the on-set of the disease, and we are currently trying to trace historical serum samples from STSS patients. As anticipated, this has turned out to be very difficult and we have so far failed to identify such samples.

Large intra-venous doses of pooled polyclonal IgG (IVIG) were reported to have beneficial effects in patients with STSS [42],[43] and in an animal model of STSS [44]. Several molecular mechanisms behind the action of IVIG have been suggested; blocking of Fc receptors, modulation of Fc receptor expression, cytokine responses and immune cell functions, neutralization of superantigens, opsonising IgG antibodies, *etc* (reviewed in [45]). IVIG is pooled from thousands of individuals and IVIG preparations contain various anti-M protein IgG antibodies [46]. Antibodies against the type-specific NH_2_-terminal region of M proteins promote killing of *S. pyogenes* in human blood, which should be a beneficial effect. However, the data of this study show that IgG antibodies binding predominantly to the central region of the M1 protein, have the potential capacity to induce a deleterious HBP release. Thus, the removal of these antibodies may enhance the therapeutic effect of IVIG. A potentially deleterious effect of certain anti-M protein IgG antibodies may also have implications for the development of an M protein-based streptococcal vaccine. However, the development of such a vaccine has lately been focused on epitopes either in the very NH_2_-terminal or in the COOH-terminal regions of the protein, which do not include the S domain (for references, see reviews [47],[48]) . The present investigation suggests that vaccines based on S domain-derived peptides, represent a risk of inducing pro-inflammatory IgG antibodies.

Sepsis in itself is a highly complex and multi-facetted syndrome [49], and STSS is usually associated with both a severe focal infection (for instance necrotizing fasciitis) and sepsis [4]. There is no doubt there are many aspects of the molecular basis for STSS pathogenesis that are still poorly understood, and the finding that a protein released from the bacterial surface forms aggregates with two human plasma proteins that activate neutrophils and induce a powerful inflammatory response, further underlines the complexity of the disease. However, a deeper molecular understanding of the patho-physiology behind the vascular leakage, which is such a pivotal symptom in STSS, should help optimize diagnosis and therapy, and perhaps identify novel therapeutic targets.

## Materials and Methods

### Reagents

Recombinant M1 protein and fragments AS and SC3 of M1 as well as IdeS and EndoS, were expressed in *E. coli* and purified as previously described [18],[19],[21]. A synthetic peptide corresponding to the S domain was produced by Biopeptide Co., Inc, San Diego, CA. Recombinant M3 and M5 was produced as previously described [50],[51]. To avoid possible LPS effects in the animal experiments, M1 protein was purified from the growth medium of the isogenic mutant MC25 strain (derived from the AP1 strain; 40/58 *S. pyogenes* strain from the World Health Organization Collaborating Centre for reference and Research on Streptococci, Institute of Hygiene and Epidemiology, Prague, Czech Republic) that lacks the membrane spanning region and secretes a soluble form of the protein [52]. Briefly, the supernatant from a MC25 culture was collected, proteins were precipitated with ammonium sulfate, dissolved in PBS, and purified on fibrinogen-coupled Sepharose. The purity of the M1 protein preparation was confirmed by SDS-PAGE analysis followed by Coomassie staining.

### Plasma absorption, IgG purification and IgG cleavage

Equal amounts of serum and protein G-Sepharose (Amersham) were incubated for 2 hours in room temperature. The mixture was centrifuged (200×*g* 1 min) and the supernatant collected. The protein G-Sepharose was resuspended in PBS, transferred to a column, washed thoroughly with PBS and then eluted with 0.1 M glycine (pH 2.0). The eluate was buffered with 1 M Tris to pH 7.5 and the absorbance read at 280 nm. To certify that all IgG had been absorbed from the supernatant, equal amounts of supernatant and protein G-Sepharose were again incubated and treated as above. The eluate from this second step did not contain any protein (no absorbance at 280 nm). IgG was purified by first desalting serum using the Zeba™ Desalt Spin Columns (Pierce) and then applied to Melon™ Gel IgG Spin Purification Kit (Pierce), as described by the manufacturer. To cleave IgG, 80 µg (2 mg/ml) of IdeS or 20 µg (1.38 mg/ml) of EndoS was added to 200 µl of serum and the solutions were incubated in 37°C for 2 hours or overnight respectively. As a control, serum without addition was treated likewise.

### Preformed protein complexes

To 50 µl of plasma was added 0.5 µg of M1 protein. The solution was left for 20 min at room temperature after which 40 µg of IdeS, 5 µg of EndoS, or buffer was added followed by incubation for 2 hours in 37°C.

### Neutrophil isolation

Human neutrophils were isolated from fresh human heparinized blood from healthy volunteers using Polymorphprep® (Fresenius Kabi) according to the manufacturer. When indicated, the neutrophils were first washed at low speed (160*g*) prior to the Polymorphprep step. Such preparations contained less than 10% platelets (free or bound to neutrophils as determined by FACS).

### Flow cytometry of neutrophil preparations

To 50 µl of isolated neutrophils were added 5 µl respectively of flourochrome-conjugated antibody CD42PerCP (BD Bioscience) and CD45FITC (Dakocytomation). After 10 minutes in room temperature the incubation was stopped by addition of 0.5% formaldehyde in PBS. Samples were analyzed using a FACSCalibur flow cytometer in linear as well as logarithmic mode. The neutrophil population was identified by its characteristics in size and granularity. Acquired cells were analyzed using Cell Quest software (Becton Dickinson)

### Determination of HBP

Neutrophils, suspended in Dulbecco's PBS, or 50 µl human blood (heparinized), diluted 10 times in Dulbecco's PBS, were incubated with various neutrophil-activating reagents for 30 min at 37°C. In the experiments with supplemented serum or IgG, serum (25 µl) or IgG (250 µg) from a responder or a non-responder was added. IgG was, when indicated, pre-treated with 0.2 µg IdeS/mg IgG (IdeS 2 mg/ml) for 2 hours in 37°C. As a control IgG without IdeS addition was treated likewise. Cells were then centrifuged 12000×*g* for 20 s and the resulting supernatants were analyzed for HBP by sandwich ELISA [53]. In order to establish the total amount HBP present, cells were lysed with 0.02% (v/v) Triton X-100 and pelleted as above.

### Blocking experiments

To 500 µl of whole blood (diluted 1∶10) 1 µl 10.1 (1 mg/ml) (Serotec®)(anti-FcγRI; CD64), 1 µl AT10 (1 mg/ml) (Serotec®)(anti-FcγRII; CD 32), 1 µl LNK16 (1 mg/ml) (Serotec®)(anti-FcγRIII; CD 16) or 1 µl of an isotype control antibody (1 mg/ml) (Ebioscience®) were added and incubated at 37°C for 20 minutes, before the samples were subjected to M1 protein stimulation. The antibodies are reported by the manufacturer to block the binding of IgG to the concomitant Fcγ-receptor and this has been demonstrated by previous studies [54]–[56].

### Determination of IgG antibodies against M1 protein

Anti-M1 IgG antibodies were detected using a modified protocol from Holm et al [41]. Microtiter plates were pre-coated with 200 µl of human fibrinogen (Sigma) (25 µg/ml) in coating buffer (15.9 mM Na_2_CO_3_ and 35 mM NaHCO_3_ pH 9.6) over night. The plates were then coated with 1.25 µg/ml M1 protein in PBST (PBS, pH 7.4; 0.05% (v/v) Tween-20) for 1 hour at 37°C, and then blocked using incubation buffer (PBST and 2% (w/v) BSA) for 20 min at 37°C. Sera diluted 1∶500 in incubation buffer were added and incubated for 1 hour, 37°C. The plates were rinsed and 200 µl of HRP-conjugated protein G (BioRad) (diluted 1∶3000) added and incubated for 1 hour at 37°C. Each incubation step was followed by a washing step. The plates were developed using chromogenic substrate as described by Tapper *et al.* [53]. Octagam (human IgG 50 mg/ml) (Octapharma) was used as a standard and the absorbance for each sera was expressed as an ELISA-index in percent relative to the absorbance of Octagam. For anti-M1 IgG antibodies in mice, sera were diluted 1∶100 and detection was made with 200 µl of HRP-conjugated goat anti-mouse IgG (BioRad) (diluted 1∶3000).

### Bactericidal assay

The bactericidal test according to Lancefield [22] was performed with the *S. pyogenes* strain AP1 in whole blood from different donors.

### Formation of protein aggregates in plasma by the addition M1 protein

M1 protein (10 µg/ml [200 nM]) was added to 500 µl of plasma (diluted 1∶10). The solution was left for 30 min at room temperature and then centrifuged 12000×*g* 1 min. The supernatant was removed and the protein aggregates in the pellet were washed three times and then resuspended in sample buffer and analyzed.

### SDS-polyacrylamide gel electrophoresis and Western blotting

Proteins were separated by 10% (w/v) SDS-PAGE in the presence of 1% (w/v) SDS [57]. Proteins were transferred onto polyvinyl chloride membranes for 60 min at 70 V. The membranes were blocked with PBS containing 5% (w/v) dry milk powder. Membranes were probed with polyclonal goat antibodies against human IgG (H+L) conjugated with alkaline phosphatase (Pierce) (dilution 1∶5000) and color was developed using Nitro Blue tetrazolium and 5-bromo-chloro-indoyl phosphate. To detect the presence of complement in the aggregates the membranes were probed with polyclonal goat IgG against complement C3 (1∶500) (purified with Protein G–Sepharose from polyclonal goat serum against complement C3 (Sigma)). The binding was visualized with rabbit anti-goat IgG conjugated to horse-radish peroxidase (Dakocytomation) (1∶1000) and developed by SuperSignal West Pico Chemiluminescent substrate (Pierce) and visualized in a ChemiDoc (BioRad). The same membrane was also probed with polyclonal rabbit antibodies against human IgG conjugated with horse-radish peroxidase (Dakocytomation) (1∶2000) and the membranes were developed using SuperSignal West Pico Chemiluminescent substrate (Pierce) and visualized in a ChemiDoc (BioRad).

### Surface plasmon resonance spectroscopy

Fibrinogen was diluted with 10 mM sodium acetate (pH 4.0) and immobilized via amine coupling in a flow cell of a CM5 sensor chip (BIAcore, Uppsala, Sweden). Immobilization level was around 1500 resonance units. A flow cell subjected to the coupling reaction without added protein was used as a control for bulk refraction index changes. Binding and dissociation were monitored in a BIAcore 2000 instrument. In control experiments for possible mass transfer effects, M1 protein was injected over the fibrinogen surface at different flow rates. No differences in initial binding rate were observed at 5 µl/min or above, indicating no mass transfer limitations. The M1 protein was injected at a determined concentration (500 nM) over the coated surface at 35 µl/min and 25°C (in running buffer: 10 mM Hepes, pH 7.5, 150 mM NaCl, 0.005% surfactant P20, and 3.4 mM EDTA). In sequence, the different antibodies were injected at an apparent M1 protein steady state binding resonance level. Antibodies were also injected directly onto the fibrinogen surface as a built-in control. The fibrinogen and blank surfaces were at the end of each experiment regenerated. This was achieved by injection of a 200 µl 2 minute-pulse of running buffer containing 2 M NaCl, followed by an extensive wash procedure.

After X and Y normalization of data, the curves from the control cell (blank) and the fibrinogen-antibody control were subtracted. The curve forms of the different antibodies were displayed in parallel using the BIA Evaluation 3.1 software (BIAcore).

### Transmission electron microscopy

M1 protein (27 µg/ml) was suspended in TBS buffer (20 mM Tris, 150 mM NaCl pH 7.4). IgG was purified as described above and applied to Zeba Desalting Spin Columns and buffered exchanged with TBS as described by the manufacturer. Five µl of M1 protein were mixed with 5 µl IgG (0.8 mg/ml) in TBS buffer and were left for 60 min in room temperature. IgG from a non-responder and a responder were used separately. The mixtures were diluted 1000 times in TBS and were analyzed by negative staining and electron microscopy as described previously [58]. 5 µl aliquots were adsorbed onto carbon-coated grids for 1 min, washed with two drops of water, and stained on two drops of 0.75% uranyl formate. The grids were rendered hydrophilic by glow discharge at low pressure in air. Specimens were observed in a Jeol JEM 1230 electron microscope operated at 60 kV accelerating voltage. Images were recorded with a Gatan Multiscan 791 CCD camera.

### Scanning electron microscopy

Fifty µl human blood (heparinized) was diluted in Dulbecco's PBS to a final volume of 500 µl, and incubated with buffer or M1 protein (1 µg/ml) for 30 min at 37°C. One hundred µl of the solution were taken off and fixed in 9.25% formaldehyde and 3.25% methanol in PBS. After 60 s of fixation 4 ml of Tyrode's buffer (137 mM NaCl, 2.8 mM KCl, 1 mM MgCl_2_, 12 mM NaHCO_3_, 0.4 mM Na_2_HPO_4_, 0.35% BSA, 10 mM HEPES and 5.5 mM Dextrose; pH 7.4) were added and left for 30 min lysing the erythrocytes. After washing in Tyrode's buffer twice, 25 µl of the lysate was applied to poly-L-lysine coated cover slips for 1 h, and subsequently fixed in 2.5% glutaraldehyde in 0.15 M sodium cacodylate, pH 7.4, (cacodylate buffer) for 30 min at room temperature. Specimens were washed with cacodylate buffer, and dehydrated with an ascending ethanol series from 50% (v/v) to absolute ethanol (10 min per step). The specimens were then subjected to critical-point drying in carbon dioxide, with absolute ethanol as intermediate solvent, mounted on aluminium holders, sputtered with 30 nm palladium/gold, and examined in a JEOL JSM-350 scanning electron microscope. The remaining 400 µl of the blood-M1 protein solution was analyzed for HBP as described above.

### Animal experiments

Male BALB/c mice (11 weeks old) were housed under standard conditions of light and temperature and fed with standard laboratory chow and water ad libitum. All animal experiments were approved by the ethics committee at Lund University. Animals were given an intraperitoneal injection of 0.3 ml of serum or PBS. After 20 hours the animals were anaesthetized with isoflurane and given an intravenous injection of 15 µg M1 per animal or 100 µl vehicle alone. Four hours post administration of M1 the mice were sacrificed. A blood sample was collected via cardiac puncture and the lungs were removed. Lung samples were fixed in 2.5% glutaraldehyde in 0.15 M sodium cacodylate, pH 7.4, (cacodylate buffer) over night at room temperature. Specimens were washed with cacodylate buffer, and dehydrated with an ascending ethanol series from 50% (v/v) to absolute ethanol (10 min per step). The specimens were then subjected to critical-point drying in carbon dioxide, with absolute ethanol as intermediate solvent, mounted on aluminium holders, sputtered with 30 nm palladium/gold, and examined in a JEOL JSM-350 scanning electron microscope.

### Immunofluorescence and confocal microscopy

A snap-frozen tissue biopsy collected from the epi-center of infection (fascia) from a patient with necrotizing fasciitis and streptococcal toxic shock syndrome caused by an M1T1 *S. pyogenes* strain (kindly provided by Prof. Donald E Low, Mount Sinai Hospital, Toronto, Canada) was cryosectioned, and the sections fixed and immunostained as previously described [11],[59]. All antibodies and fluorochromes were diluted in PBS-saponin supplemented with Aurion BSA-c. Staining for fibrinogen was obtained by incubation overnight with purified rabbit anti-fibrinogen antibodies (Dakocytomation) diluted to a concentration of 3 µg/ml, followed by a 30 min incubation with biotinylated goat-anti-rabbit IgG (diluted 1∶500; Vector Laboratories, Burlingame, CA), and subsequent addition of streptavidin conjugated Alexa Fluor 633 (diluted 1∶500; Molecular Probes, Eugene, OR, USA). The M1 protein staining was achieved by incubation overnight with biotinylated purified polyclonal rabbit anti-M1 protein antibodies at a concentration of 2 µg/ml, followed by streptavidin conjugated Alexa Fluor 546 (diluted 1∶500; Molecular Probes). Immunoglobulins were detected by incubation with protein L from the anaerobic bacterial species *Finegoldia magna*, a protein with high affinity for Ig light chains [29]. A biotinylated protein L was used at a concentration of 0.5 µg/ml and detected by streptavidin conjugated Alexa Fluor 488 (diluted 1∶600; Molecular Probes). Vectashield supplemented with dapi (Vector Lab.) was used as mounting media. The co-localization of all three markers was assessed in a triple staining. The staining pattern (i.e. area and intensity of positive signals) for each marker was highly similar in single and triple stainings. Hence, non-specific signals due to interactions between antibodies or with the Fc-binding portions of the M-protein could be excluded. Sections stained in an identical manner, except for omission of primary antibody, were used to set the threshold of the laser. This threshold level was then applied for the entire analysis; thereby controlling for autofluorescence in the tissue. For evaluation, the Leica confocal scanner TCS2 AOBS with an inverted Leica DMIRE2 microscope was used.

### Statistical methods

Differences between various treatments of blood samples were assessed with respect to release of HBP first with ANOVA or ANOVA on ranks, and if a difference was proven the different groups were compared against the control sample with Dunnett's 2 sided test or Mann Whitney U test. P-values of 0.05 or less were regarded as significant.
